# DnaJ molecules as potential effectors in *Meloidogyne arenaria*. An unexplored group of proteins in plant parasitic nematodes

**DOI:** 10.1080/19420889.2019.1676138

**Published:** 2019-10-16

**Authors:** Rosita Grijalva-Mañay, Carmen Dorca-Fornell, Wladimir Enríquez-Villacreses, Gabriela Miño-Castro, Ricardo Oliva, Valeria Ochoa, Karina Proaño-Tuma, Vinicio Armijos-Jaramillo

**Affiliations:** a Department of Life Sciences, Laboratory of Plant Biotechnology, Armed Forces University ESPE, Sangolquí, Ecuador; b Genetics and Biotechnology, International Rice Research Institute (IRRI), 4031 Laguna, Philippines; c Carrera de Ingeniería en Biotecnología, Facultad de Ingeniería y Ciencias Aplicadas, Universidad de Las Américas, Quito, Ecuador; d Grupo de Bio-Quimioinformática, Universidad de Las Américas, Quito, Ecuador

**Keywords:** Nematode, effector, DnaJ protein, M. arenaria, secreted, nuclear localization signal

## Abstract

Plant pathogenic organisms secrete proteins called effectors that recognize, infect and promote disease within host cells. Bacteria, like *Pseudomona syringae*, use effectors with DnaJ function to disrupt plant defenses. DnaJ proteins (also called Hsp40) are a group of co-chaperone molecules, which assist in the folding of proteins. Despite the described role of DnaJs as effectors in several groups of pathogens, this group of proteins has never been correlated with the infection process in plant parasitic nematodes. In this study, we analyze the importance of DnaJ for plant parasitic nematodes. To do that, we compare the number of DnaJ proteins in nematodes with different lifestyles. Then, we predict the secreted DnaJ proteins in order to detect effector candidates. We found that *Meloidogyne* species have more secreted DnaJs than the rest of the nematodes analyzed in the study. Particularly, *M. arenaria* possess the highest proportion of secreted DnaJ sequences in comparison to total DnaJ proteins. Furthermore, we found in this species at least five sequences with a putative nuclear localization signal, three of them with a serine rich region with an unknown function. Then, we chose one of these sequences (MG599854) to perform an expression analysis. We found that MG599854 is over-expressed from 3 days post inoculation onwards in tomato plants. Moreover, MG599854 seems to be enough to produce cell death in *Nicotiana benthamiana* under transient expression conditions. In concordance with our results, we propose that DnaJ proteins are a potential source of effector proteins in plant parasitic nematodes.

## Introduction

Plant parasitic nematodes constitute one of the most important groups of plagues around the world. The losses produced by these animals are estimated at between 80 to 175 billion US dollars per year in agricultural crops [1]. Among nematodes that produce root knots (RKN), *Meloidogyne* spp. are particularly important due to their ability to attack a wide range of crops [2]. Virtually any higher plant could be host to at least one species of this genus [3]. Additionally, *Meloidogyne* was selected as the most important nematode group for phytopathologists [4]. There are over 100 species of *Meloidogyne* distributed worldwide, with the greatest economic damage attributed to the four most common species: *M. incognita, M. javanica, M. arenaria* and *M. hapla* [5,6].

During plant-parasite interactions, many nematode genes are induced, contributing to the onset of the disease [7]. These genes encode proteins called effectors that are secreted through the stylet for modifying the structure and functions of the host cells and facilitating the infection [8]. Several effectors have been already characterized in nematodes, such as SPRYSEC-19 [9] and GrUBCEP12 [10] in *Globodera rostochiensis*; HsCLEB in *Heterodera schachtii* [11]; and HgCLE in *Heterodera glycines* [12,13]. In the genus *Meloidogyne*, at least 10 effectors have been characterized [14], for example, calreticulin Mi-CRT from *Meloidogyne incognita* and the MeTCPT of *Meloidogyne enterolobii*, which have been demonstrated to suppress plant defenses directly [9,10,15–17].

The effector HopI1 is present in the phytopathogenic bacteria *Pseudomona syringae* [18]. This protein remodels the thylakoid structure of the chloroplast to suppress the salicylic acid accumulation necessary to activate pathways against bacteria [19]. HopI1 have a J domain (also called DnaJ domain) that is essential in order for it to interact with HSP70 chaperone proteins. DnaJ proteins (also known as HSP40s) constitute a family of conserved co-chaperone molecules that ease the folding of proteins [20]. However, other functions have been detected for this kind of molecules, for example, Cwc23 of *Saccharomyces cerevisiae* is involved in pre-mRNA splicing. These proteins can act without the presence of a J domain [21]. In addition, ERdj5 is able to activate the endoplasmic reticulum calcium pump without interacting with a HSP70 protein [22].

Structurally, DnaJ proteins consists of a N-terminal conserved domain (called ‘J’ domain) with roughly 70 amino acids, a glycine rich region (‘G’ domain) with roughly 30 residues, a central domain with 4 repetitions of a CXXCXGXG (‘CRR’ domain) motif, and a C-terminal region of 120 to 170 residues. The characteristic region of this group is the J domain, which interacts and regulates HSP70 using the conserved motif HPD [20]. Type I DnaJ proteins have four regions (J domain, G domain, CRR domain and C-terminal region), type II lacks the CRR region and type III proteins have only the J domain and this can be located anywhere in the molecule. A special type (type IV) was created for the type III proteins with HXD motif instead HPD. This type was reported in parasites and there no homologues in humans [23].

DnaJ have been associated with several infective processes in different organisms. In *Streptococcus pneumoniae*, SpDnaJ is essential for virulence and colonization [24]. In *Vibrio tapetis* and *Legionella dumoffii*, DjlA showed an important role in cytotoxicity and intracellular growth, respectively [25,26]. In *Plasmodium falciparum*, DnaJ proteins might be involved in host cell modification and in the maintenance of exported proteins before their final destination in the host cell [27,28]. In nematodes, DnaJ proteins have not been related with infective mechanisms; instead, in *C. elegans*, several Hsp40 molecules were associated with the regulation of the locomotor circuit and also with homeostasis and integrity of mitochondria [29,30]. In light of these antecedents, the role of DnaJ in nematode pathogenicity is more than probable and should be explored.

In this study, a bioinformatic analysis was carried out in order to attempt to identify the DnaJ copy number in several nematodes. Furthermore, following the initial results, we studied the domain structure and classification of *M. arenaria* DnaJ proteins. Then, we identified putative effectors according to the domain structure and predicted subcellular localization. Finally, we chose one of the candidates to perform transient expression experiments and observed the effect *in planta*. To the best of our knowledge, this is the first time that DnaJ proteins have been analyzed in plant parasite nematodes and the first time that a DnaJ sequence has been proposed as an effector in this group of organisms.

## Materials and methods

### Proteomic search and domain determination of Dnaj proteins in nematodes

The predicted proteins of 16 nematodes with different lifestyles were downloaded from WormBase (https://parasite.wormbase.org/ftp.html). From these, duplicated sequences (with identical residues) were dismissed from the dataset. The proteomes were used as a database to perform a PSI BLAST search [31] (3 iteration, e-value 10–5), using different queries (Table 1). For all PSI BLAST results, the presence of the J domain (InterPro: IPR001623) and the HXD motif were verified before accepting the proteins as members of the DnaJ family. InterProScan 5 [32] was used to identify the presence of the characteristic DnaJ domains in the dataset (J domain, CRR domain, and the C-terminal domain), as well as other domains not related to DnaJ proteins. To predict the G/F domain (a characteristic domain of several DnaJ proteins), a hidden Markov model was created with HMMER [33] using the alignment of the DnaJ type I family of *E. coli* TIGR02349 (TIGRFAMs database [34]) as input. As the G/F rich regions lack any secondary structures in contraposition to the J domain, which is formed by several alpha helices [35], the secondary structure of the whole protein dataset was predicted using the Garnier program within the EMBOSS package [36]. Prior to marking any regions as G/F rich domains, the protein zones predicted by the hidden Markov model as G/F rich domains were verified by the lack of alpha helix conformation in the secondary structure prediction and the abundance of G and F. The CRR domain and the C-terminal domain are included in the InterPro database and therefore, InterProScan directly performed the prediction of these domains.

**Table 1.** Nematode species used in this study and the number of predicted DnaJ by proteome.

Species	Lifestyle	Protein number	BioProject^a^	Query^b^	Number of DNAj	Number of secreted DNAj^c^
*Caenorhabditis brenneri*	Free-living	30,017	PRJNA20035	G0N2B2_CAEBE	36	4
*Caenorhabditis briggsae*	Free-living	23,913	PRJNA10731	A8XMW5_CAEBR	36	5
*Caenorhabditis elegans*	Free-living	28,196	PRJNA13758	Q9XWE1_CAEEL	34	3
*Caenorhabditis japónica*	Free-living	35,013	PRJNA12591	A0A2H2HXM1_CAEJA	33	4
*Caenorhabditis remanei*	Free-living	31,235	PRJNA53967	A0A260YRA3_CAERE	26	3
*Pristionchus pacificus*	Free-living	25,204	PRJNA12644	A0A2A6BSD8_PRIPA	24	3
*Ascaris suum*	Animal parasite	46,912	PRJNA62057	F1KVF4_ASCSU	72	4
*Brugia malayi*	Human parasite	14,293	PRJNA10729	A0A0K0J867_BRUMA	36	3
*Loa loa*	Human parasite	15,319	PRJNA37757	A0A1I7VI53_LOALO	31	3
*Wuchereria bancrofti*	Human parasite	13,053	PRJEB536	A0A1I8EK19_WUCBA	25	1
*Trichinella spiralis*	Human parasite	16,067	PRJNA257433	A0A0V1B3E5_TRISP	20	3
*Bursaphelenchus xylophilus*	Plant parasite	17,652	PRJEA64437	A0A0K0F543_STRVS	28	2
*Globodera pallida*	Plant parasite	16,312	PRJEB123	A0A183CPU2_GLOPA	36	1
*Meloidogyne hapla*	Plant parasite	14,378	PRJNA29083	A0A1I8AZ83_MELHA	32	4
*Meloidogyne incognita*	Plant parasite	21,673	PRJNA340324	A0A2L0VY59_MELAR	47	8
*Meloidogyne arenaria*	Plant parasite	98,472	PRJEB8714	A0A2L0VY59_MELAR	141	32

^a^BioProject ID where the proteome was extracted

^b^Uniprot ID of the protein used as a query in the PSI BLAST analysis

^c^The number of secreted proteins was calculated according to the predictions of SignalP

### *M.arenaria* Dnaj proteins tree reconstruction

*M. arenaria* DnaJ predicted proteins were aligned with MAFFT v7 [37]. The multiple sequence alignment was edited manually. The maximum likelihood tree was reconstructed with FastTree 2.1.5 [38] and supported with FastTree support value.

### Subcellular localization and nuclear localization signal prediction

SignalP 4.0 [39] and TMHHM 2.0 [40] programs were used to predict the putative subcellular space occupied by the DnaJ candidates. Sequences with signal peptides and without transmembrane regions were annotated as extracellular proteins. WoLF PSORT v0.2 [41] was also used to predict the subcellular localization of proteins annotated as non-secreted. Additionally, the nuclear localization signal was predicted for the secreted proteins dataset using NucPred [42], cNLS [43] and NLStradamus [44]. Putative repetitive regions were predicted with RADAR [45]

### *Meloidogyne arenaria* inoculum preparation

*Meloidogyne arenaria* inoculum was collected from *Hypericum perforatum* plants showing a high rate of root galling. These plants were provided by the Hilsea Esmeralda Breeding Farm, which is located at 0º13ʹ0 “S – 78º58ʹ2” at an altitude of 2219 meters above sea level in the town of El Quinche near Quito in Ecuador. In order to verify the species collected in the root galls, following a microscopic identification, SCAR primers were amplified to differentiate between *M. arenaria, M. incognita* and *M. javanica*. The primers described by Zijlstra, Donkers-Venne & Fargette [46] were used for this purpose. We used a verified genomic DNA of *M. incognita* as a positive control for the SCAR amplification. The Phyto and Zoosanitary Regulation and Control Agency of Ecuador (Agrocalidad) kindly provided the DNA.

The inoculum preparation was conducted in two parts: the first step was the removal of nematode eggs from infected plants by the method described by Hussey & Barker [47]. The second step was to place the extracted eggs in a Petri dish and cover with a plastic lid. This was allowed to stand for 4 to 7 days with water level checked every two days to prevent the eggs drying. The inoculum was aired at room temperature by using a pump, until use. Infective stage J2 was identified using an optical microscope.

### Plant growing and nematode infection conditions

The Flora Dade variety of *Solanum lycopersicum* was maintained under controlled conditions in a greenhouse, at 20**°**C and long-day conditions (16 hours of light and 8 hours of darkness) and then infected with the inoculum of *Meloidogyne arenaria*. We used approximately 5,000 J2 stage larvae to establish the infection. Nematode dormancy was broken 72 hours after plant inoculation, followed by root penetration and infection. For this reason, we excluded 0 days post inoculation (dpi) from the analysis and we started to evaluate the plants 3 dpi.

*Nicotiana benthamiana* was also maintained under controlled conditions in a greenhouse at 22ºC and long-day conditions (16 hours of light and 8 hours of darkness).

### RNA extraction and cdna synthesis

RNA from root-infected samples were extracted using Trizol® reagent (Invitrogen Thermo Fisher, US) and then treated with DNAse. cDNA synthesis was performed using the SuperScript III kit Reverse Transcriptase (Invitrogen Thermo Fisher, US) according to the manufacturer’s protocol.

Samples for RNA extraction were obtained 3, 5, 7, 9, 14 and 21 dpi.

### RT-PCR and sequencing

Amplification of the MG599854 gene was performed by RT-PCR with Platinum Taq Polymerase enzyme (Invitrogen, Thermo Fisher, US). For primer design, we used the Primer3 V.0.4.0 [48] and M.Arenaria_Scaff164g004450 transcript sequence as a template (Bioproject ID: PRJEB8714, sample: ERS1696677 [49]). SignalP was used to avoid the generation of primers on a signal peptide region. The primer-obtained sequences were the following: 5ʹ GAGTCTATACCTAACTACTACG 3ʹ and 5ʹ TCAAATACCGAAATCTGT 3ʹ. Finally, nematode *GAPDH* (Glyceraldehyde-3-Phosphate Dehydrogenase) (accession number U7257.1) was employed as an internal control to assess the quality of the synthesized cDNA. *GAPDH* primers were also designed by Primer3 V.0.4.0 and their sequences were the following: 5ʹ GGTTACAGTTCCCGTGTGATTG 3ʹ and 5ʹ AGACCACTCCAGGCCCACAAAA 3ʹ. Furthermore, cleavage sites of the restriction enzymes NotI (5ʹ … … 3ʹGCGGCCGC) and ClaI (ATCGAT 5ʹ … … 3 ‘) were added to the 5ʹ end of the forward and reverse primers of each gene, which are necessary for subsequent cloning assays.

Before obtaining the sequences of the amplified products, they were purified by the QIAquick PCR purification kit (Qiagen Corporation, US). The sequences were sequenced and analyzed.

### Agro-infiltration and transient expression of the MG599854 gene in *Nicotiana benthamiana* plants

The cloning vector *pGR106* was used to insert the MG599854 gene. *pGR106* uses the *CaMV35S* (cauliflower mosaic virus) as a promoter and the neomycin phosphotransferase (that confers kanamycin resistance) as a selection gene. The vector also has restriction sites for ClaI/AscI/NotI/SalI enzymes and RB and LB borders of the *Ti* plasmid of *Agrobacterium tumefaciens*, which are necessary for expressing the target genes. Both the vector and the amplified fragments were digested with ClaI and NotI enzymes, which yielded fragments of cohesive 5ʹ and 3ʹ ends. Ligation was performed using a T4 ligase enzyme. *Agrobacterium tumefaciens GV3101* was grown in Luria-Bertani medium enriched with rifampicin and tetracycline (LB) at 28°C. The ligation products obtained (PGR106 vector + MG599854 gene) were cloned into electrocompetent bacteria using the Eppendorf electroporator Eporator® by V1.01 (Cole-Parmer Instrument Company, US). A rapid extraction of bacterial DNA was performed and positively transformed bacteria were evaluated by colony PCR using the primers PVX-F 5´AATCAATCACAGTGTTGGCTTGC 3´ and PVX-R 5´AGTTGACCCTATGGGCTGTGTTG 3´.

Positively transformed *Agrobacterium tumefaciens* bacteria were inoculated individually in *Nicotiana benthamiana* leaves (10 replicates). As a positive and negative control, *Agrobacterium tumefaciens GV3101* strains transformed with the *INF1* gene and the *GFP* gene were used, respectively. The Sainsbury Laboratory in England kindly provided the *Agrobacterium tumefaciens GV3101* transformed with INF1 and GFP genes.

## Results

### Dnaj proteins in nematodes

We explored the characteristics and copy number of DnaJ proteins (also called Hsp40) in the proteome of several nematodes with different lifestyles. In that sense, we used a search based on PSI BLAST using each proteome as a database and different DnaJ proteins as the query. Then, we predicted signal peptides to infer the putative secreted proteins (Table 1). We calculated the ratio of DnaJ and secreted DnaJ proteins with the total number of proteins predicted for each species. The distribution of these ratios did not return any outlier for the total number of DnaJ sequences in nematodes proteomes. However, *Meloidogyne* species had a higher ratio of secreted DnaJ than the other nematodes analyzed in this study (Supplementary table 1). In addition, *M. arenaria* had the highest proportion of secreted DnaJ sequences in comparison to the total number of predicted DnaJ proteins (Supplementary figure 1).

### *M. arenaria* Dnaj proteins

Motivated by the higher proportion of secreted DnaJ proteins in *M. arenaria*, we decided to analyze this economically relevant species in depth. Thus, we predicted motifs and classified 141 proteins annotated with J domains.

To classify the DnaJ proteins, we used the Rug and Maier [23] criteria (type I, II, III and IV DnaJ proteins). We determined the presence of three other domains that are usually present in DnaJ proteins: a G/F rich domain, a Cysteine Rich Region (CRR) domain, and a C-terminal domain (see experimental procedures section). After domain prediction, 5 type-I DnaJ proteins, 6 type-II DnaJ proteins, 23 type-III DnaJ proteins and 1 type-IV sequences were identified. Additionally, five sequences were annotated with the J domain and the C terminal domain, without the presence of GF-rich and CRR domains. These sequences do not fit with the Rug and Maier classification criteria. Taking into account all the domains predicted, twenty-three different patterns were observed in the dataset of 141 J domain/HXD containing proteins (Supplementary table 2).

**Figure 1.**
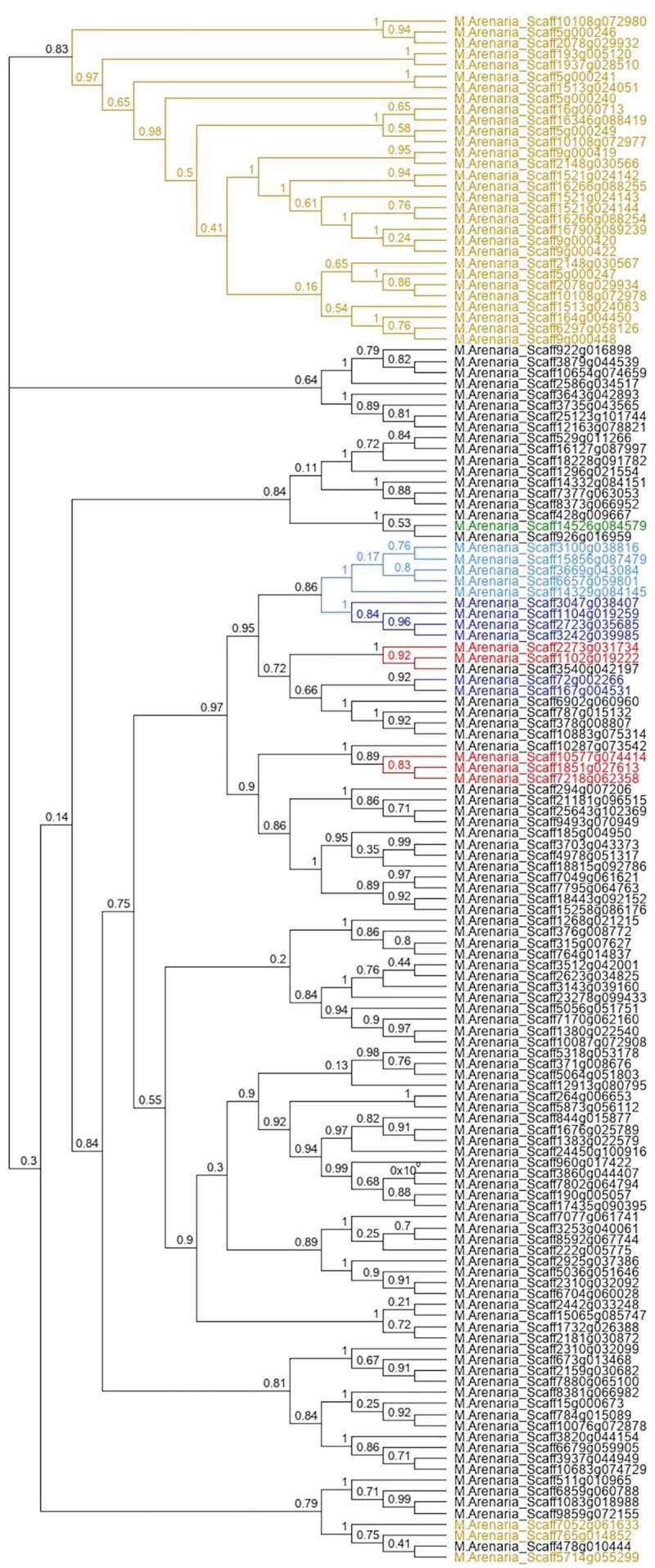
Maximum likelihood tree reconstructed with DNAj proteins found in *Meloidogyne arenaria*. The accession numbers highlighted in red correspond to the members of the DNAj type I proteins, the blue labels correspond to DNAj type II proteins, black labels to DNAj type III proteins and green labels to DNAj type IV proteins, in concordance with Rug and Maier [23]. Blue light labels correspond to DnaJ proteins with J domains and DnaJ C-terminal domains and yellow labels correspond to the putative secreted proteins. The tree was supported by FastTree support value.

A maximum likelihood tree was used to deduce the phylogenetic relationships between DnaJ proteins (Figure 1). All sequences of type-I and II were clustered in the same branch with the proteins that only had J and C terminal domains. Furthermore, almost all the proteins predicted with a signal peptide shared the same cluster in the tree.

The most intriguing DnaJ proteins observed in *M. arenaria* were the predicted with signal peptide and nuclear localization signal. Moreover, in three of these sequences, we identified a repetitive serine rich domain (S-rich). In fact, the protein M.Arenaria_Scaff164g004450 has only one of these domains while M.Arenaria_Scaff6297g058126 has four. M.Arenaria_Scaff9g000448 has three repetitive serine rich domains with a slightly different repetition nucleus (S-rich2), plus 2 S-rich repetitions (Supplementary table 2). The repetition pattern of the S-repeat is [PFLS]-X-[MT]-S-G-[ED]-S-[ST]-X-S-S-[EVDS]-[GR]-X and the sequence of the S-repeat2 is ASTTSGDSSTSSEGSSSSSVG. In addition, we predicted putative nuclear localization signals (NLS) in secreted DnaJ proteins. The three sequences with S repetitive domains showed NLS with a high score in cNLS mapper, a program that predicts specific NLS for importins (alpha and beta). The NLS predicted for these proteins was a section of 10 aa in length (S-K-R-K-K-A-H-T-[ND]-F). Additionally, two other secreted proteins without S repetitive domains were also predicted with a NLS (M.Arenaria_Scaff1937g028510 and M.Arenaria_Scaff193g005120). These proteins obtained high scores in NucPred predictor and M.Arenaria_Scaff1937g028510 was predicted with a NLS also by the NLStradamus program. No other secreted DnaJ sequence was predicted with a NLS, at least not with high scores. In addition to *M. arenaria*, from the other 15 nematodes species analyzed in this study, only 6 presented one secreted DnaJ with NLS and only *M. incognita* showed two (data not shown). This observation highlights the tendency of *M. arenaria* to duplicate DnaJ genes.

We argue that secreted proteins with repetitive regions and NLS could have a role to play in the pathogenicity of nematodes, maybe by acting as effectors. In that sense, we decided to explore the effect of M.Arenaria_Scaff164g004450 *in planta*. To do that, we isolated *M. arenaria* from infected *Hypericum perforatum* plants. Before performing experiments with the isolated nematode, we verified the species by microscopic observation and then with a species-specific SCAR marker (see methods section) (Supplementary figure 2).

### Sequencing of *M.arenaria_scaff164g004450* in *M. arenaria* local isolate

**Figure 2.**
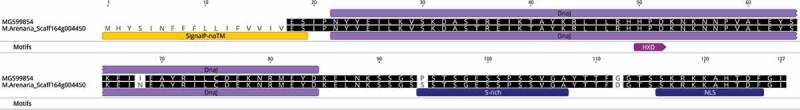
Alignment between M.Arenaria_Scaff164g004450 and MG599854. Domain annotations are highlighted behind the alignment.

To further validate the presence of *M.Arenaria_Scaff164g004450* in the local *M. arenaria* isolate, we sequenced the coding region of the gene from cDNA. We obtained a 346 bp sequence (Genbank ID: MG599854), 97.9% identical to *M.Arenaria_Scaff164g004450* cDNA. At amino acid level, these sequences differ in three residues, one in the DnaJ domain, another in the S-rich domain and the last before the NLS (Figure 2).

### MG599854 is induced during root infection

**Figure 3.**
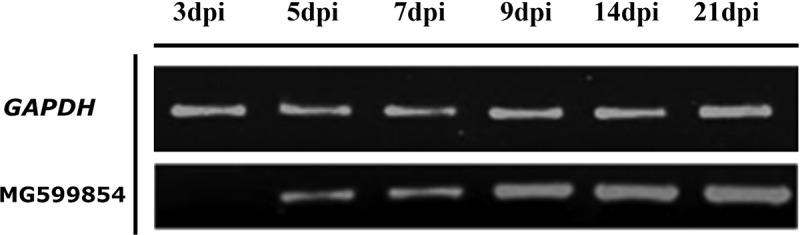
MG599854 gene expression on tomato-root samples infected with *Meloidogyne arenaria* at 0, 3, 5, 7, 9, 14, and 21 dpi (days post infection). MG599854 expression is visible from 5 dpi and progressively increases until 25 dpi. *GADH* gene was used as internal control.

To determine the behavior of MG599854 during colonization of tomato roots, we analyzed transcript accumulation of this gene over a time period by means of an infection experiment using semi-quantitative RT-PCR. The gene expression of MG599854 was up-regulated at 5 dpi (days post inoculation) and continuously increased to 21 dpi. No expression was detected at 3 dpi (Figure 3).

### Transient expression of MG599854 produces cell death in *Nicotiana benthamiana* leaves

**Figure 4.**
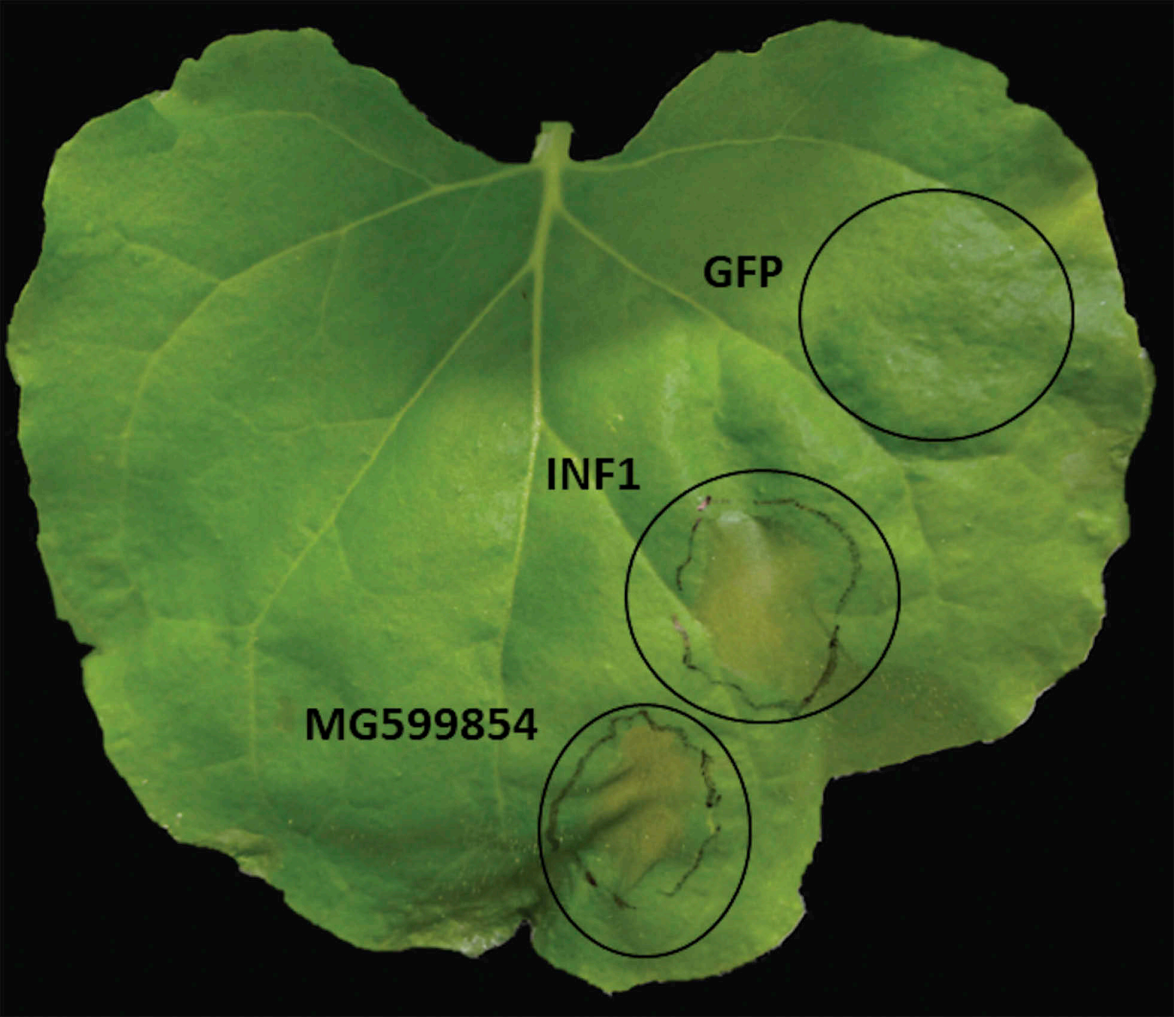
Ectopic expression of MG599854 by agroinfiltration technique in *Nicotiana benthamiana* leaves assessed at 3 days post inoculation (dpi). *INF1* and *GPF* genes are positive and negative controls, respectively. Cell death is visible in MG599854 and *INF1* gene expression (marked with a circle on the leaf).

In order to shed light on the function of MG599854, we agro-infiltrated the coding region of the gene in *N. benthamiana*. We observed that the ectopic expression of MG599854 induced cell death in 80% of the infiltrated leaves after 3 dpi. The same result was obtained with the effector gene *INF1* of *Phytophthora infestans*, used as a positive control. In the transient expression of GFP (negative control), no visible response was observed (Figure 4).

## Discussion

The aim of this study was to explore the relevance that DnaJ proteins could have in the plant parasitic lifestyle of nematodes. Following the data, we found several DnaJ sequences in *M. arenaria* with characteristics usually regarded in effectors. One of these (MG599854) even induced cell death *in planta* under transient expression conditions.

The number of DnaJ proteins predicted by our analysis for nematodes (between 20 and 141) are comparable with estimations in *A. thaliana* [50], *S. cerevisie* [51], *D. melanogaster* [52] and even with the one obtained by the *C. elegans* Sequencing Consortium [53]. No obvious difference between lifestyles was observed in the ratio of DnaJ copy number among nematodes proteomes. However, members of the *Meloidogyne* species were the organisms with the highest ratio of secreted DnaJ sequences. In fact, the ratio of secreted DnaJ versus the total number of DnaJ was also higher in *Meloidogyne* species and remarkably high in *M. arenaria* (Supplementary table 1). These data suggest a relevant role of secreted DnaJs in the evolution of the genus and could be relevant in the parasitic behavior of these nematodes. In the human parasite *Plasmodium falciparum*, the secreted DnaJ proteins seem to have expanded across their evolution [54]. This expansion has been correlated with the parasite’s ability to invade and remodel human cells [55]. Despite the abundance of the secreted DnaJ proteins in *Meloidogyne* species, other plant parasitic species like *Bursaphelenchus xylophilus* and *Globodera pallida* do not have high ratios of secreted DnaJ proteins. Actually, *Globodera pallida* has the lowest ratio of all species evaluated. This suggests that the high proportion of secreted DnaJ is characteristic of *Meloidogyne* and not of all plant parasitic nematodes.

The four classes of DnaJ proteins (according to the Rug and Maier [23] criteria) were identified in the proteome of *M. arenaria*. This includes a type IV protein (M.Arenaria_Scaff14526g084579), a group of proteins with a motif HXD in the J domain that was identified initially in *Plasmodium falciparum* proteome. Type IV proteins were identified in several apicomplexan organisms without homologues in humans [55]. To the best of our knowledge, this is the first time that a DnaJ type IV protein has been identified in nematodes.

The tree reconstruction performed on *M. arenaria* DnaJ proteins showed a level of clustering between types. Particularly, sequences classified as type I, II and their variants (like proteins with N and C terminal DnaJ domains only) share the same branch in the tree. In addition, most proteins with signal peptides were clustered in the same branch (Figure 1). The association of secreted proteins was also observed in the phylogenetic analysis of *A. thaliana* DnaJ proteins [50]. A similar pattern was observed in Hsp40 proteins of *P. falciparum*, clustering the majority of type I and II proteins in the same branch (including secreted sequences). The secreted type III sequences were also clustered in the same branch [55]. A remarkable difference to *P. falciparum* was that no secreted protein was identified as type I or II in *M. arenaria*. The phylogenetic pattern of both studies suggests that type I and II share a recent ancestor and/or are conserved for evolutionary pressure. However, type III proteins seem to have an uncorrelated phylogenetic distribution with the domain content (see Figure 1 and Supplementary table 2). According to Rug and Maier [23] and Ajit Tamadaddi and Sahi [56], type III DnaJ proteins have diverse and in several cases unknown functions, whereas type I and II (despite their variable functions) have the conserved role of binding with HSP70 to deliver the substrate. A paraphyletic origin of type III proteins by domain shuffling could explain the phylogenetic pattern observed for this type. However, the secreted proteins (all annotated as type III) clustered in the same branch of the DnaJ tree suggest the expansion of this group from a common ancestor. At the same time, this expansion indicates a relevant role for secreted DnaJ in *M. arenaria* adaptation.

We detected at least five secreted DnaJ proteins with a predicted nuclear localization signal (NLS). In sedentary plant parasitic nematodes, it has been proposed that these kinds of molecules have important roles in host cell remodeling, establishing the feeding sites and counteracting plant defenses [57]. Several effectors have been characterized with NLS in *Meloidogyne* spp. For example, the effector MJ-NULG1a of *M. javanica* was predicted with two NLS and then confirmed with immunolocalization [58]. The effector MiISE6 of *M. incognita* is secreted into the plant and possesses two NLSs. The nuclear localization was verified by means of the fusion of the gene with GFP and the posterior detection by means of confocal microscopy during transient expression in *N. benthamiana* [59]. For the effector 7H08 of *M. incognita*, the NLSs were verified experimentally, regarding the protein inside the host nucleus [60].

Three of the five secreted DnaJ sequences with NLS found in *M. arenaria* possess one or more serine rich regions, two of them in tandem repetitions. The repetitive tandem or no-tandem regions are constantly observed in effectors of plant-associated organisms [61]. However, serine rich repetitive regions in effectors have not been reported to this date. Despite the reported functions of repetitive regions in effectors (localization, stability, molecule interactions), we do not have any previous reference of the role that a repetitive serine region could play. To explore the potential role of this kind of molecule in pathogenesis, we decided to explore the effect of M.Arenaria_Scaff164g004450 (sequence with only one serine rich region) during infection *in planta*. To do that, we sequenced a copy of this gene from a local isolate of *M. arenaria*. The result was almost identical to *M.Arenaria_Scaff164g004450* and was codified as MG599854 by GenBank. MG599854 was over-expressed at 5 dpi and continuously increased until 21 dpi in *Solanum lycopersicum* infected plants. According to Curtis [62], many genes involved in nematode parasitism that are related to the formation of giant cells are expressed during the early stages of infection. This suggests that the gene could be involved in the initial processes of infection. Additionally, transient expressions were performed in *N. benthamiana* plants. We used three different genes for the experiment: the reporter gene *GFP*, the effector gene of *P. infestans INF1* and MG599854. GFP did not produce cell death while MG599854 did. This observation shows that the cell death observed was not produced by the transient expression itself. The cell death produced by INF1 was similar in size and shape to the MG599854 agroinfiltration (Figure 4). Effector proteins often disrupt the host immunity, producing phenotypes reaching from chlorosis to necrosis. The same behavior was observed in several effectors in transient expression experiments [63]. In that sense, MG599854 could work as an effector protein, probably translocated to the plant cell nucleus.

Despite the fact that we demonstrate the expression of a DnaJ protein during infection and the cell death produced by transient expression, this does not show an effector function for this group of proteins. An *in vivo* localization experiment together with the construction of J domain mutants (particularly the HPD site) and NLS determination needs to be performed to validate the predicted secreted proteins with nuclear localization and to determine the importance of these molecules in the infection process.

In this study, we explored the impact of DnaJ proteins in nematodes and particularly in the plant pathogen nematode *M. arenaria*. For *Meloidogyne* species, we explored for the first time a potential role of DnaJ proteins as effectors. We found a large number of secreted proteins in *Meloidogyne* species and the highest one in *M. arenaria*. Additionally, in this species we predicted secreted DnaJ proteins with NLS. Experimentally, we demonstrated that at least one of these NLS sequences is expressed during the infection process and is able to cause cell death during transient expression. This research attracts attention in the study of DnaJ proteins in plant parasitic nematodes and reveals the importance that this kind of protein could have in the infection process. For instance, DnaJ proteins can be used to identify previously unknown defense components, whose engineering may lead to novel approaches for creating disease-resistant plants.

## Supplementary Material

Supplemental Material
