# Supplementary material for: DnaJ molecules as potential effectors in Meloidogyne arenaria. An unexplored group of proteins in plant parasitic nematodes
Source: Commun Integr Biol. 2019 Oct 16;12(1):151–61. doi: 10.1080/19420889.2019.1676138 (PMC6802931; doi:10.1080/19420889.2019.1676138)
Supplement: Supplemental Material [file kcib-12-01-1676138-s001.pdf]

Supplementary table 1. Ratios between DnaJ and the total number of proteins annotated in each nematode genome project

Species	Lifestyle	DnaJ/complete proteome (x100)	Secreted DnaJ/complete proteome (x100)	Secreted DnaJ/total DnaJ
Meloidogyne incognita	Plant parasite	0.217	0.037	17.02
Meloidogyne arenaria	Plant parasite	0.143	0.032	22.69
Meloidogyne hapla	Plant parasite	0.223	0.028	12.5
Brugia malayi	Human parasite	0.252	0.021	8.33
Caenorhabditis briggsae	Free-living	0.151	0.021	13.89
Loa loa	Human parasite	0.202	0.020	9.67
Trichinella spiralis	Human parasite	0.124	0.019	15
Caenorhabditis brenneri	Free-living	0.120	0.013	11.11
Pristionchus pacificus	Free-living	0.095	0.012	12.5
Caenorhabditis japónica	Free-living	0.094	0.011	12.12
Bursaphelenchus xylophilus	Plant parasite	0.159	0.011	7.14
Caenorhabditis elegans	Free-living	0.121	0.011	8.82
Caenorhabditis remanei	Free-living	0.083	0.010	11.54
Ascaris suum	Animal parasite	0.153	0.009	5.56
Wuchereria bancrofti	Human parasite	0.192	0.008	4
Globodera pallida	Plant parasite	0.221	0.006	2.77

Supplementary table 2. Classification of *M. arenaria* DnaJ proteins by domain structure

Protein ID	Domain structure[a]
M.Arenaria_Scaff25643g102369<br>M.Arenaria_Scaff25123g101744<br>M.Arenaria_Scaff24450g100916<br>M.Arenaria_Scaff23278g099433<br>M.Arenaria_Scaff21181g096515<br>M.Arenaria_Scaff18443g092152<br>M.Arenaria_Scaff17435g090395<br>M.Arenaria_Scaff16346g088419<br>M.Arenaria_Scaff15258g086176<br>M.Arenaria_Scaff15065g085747<br>M.Arenaria_Scaff14526g084579<br>M.Arenaria_Scaff12913g080795<br>M.Arenaria_Scaff10287g073542<br>M.Arenaria_Scaff10076g072878<br>M.Arenaria_Scaff9493g070949<br>M.Arenaria_Scaff8592g067744<br>M.Arenaria_Scaff8381g066982<br>M.Arenaria_Scaff7802g064794<br>M.Arenaria_Scaff7795g064763<br>M.Arenaria_Scaff7077g061741<br>M.Arenaria_Scaff7049g061621<br>M.Arenaria_Scaff5873g056112<br>M.Arenaria_Scaff3937g044949<br>M.Arenaria_Scaff3860g044407<br>M.Arenaria_Scaff3512g042001<br>M.Arenaria_Scaff3253g040061<br>M.Arenaria_Scaff3143g039160	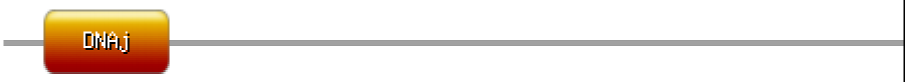

M.Arenaria_Scaff2925g037386<br>M.Arenaria_Scaff2623g034825<br>M.Arenaria_Scaff2310g032099<br>M.Arenaria_Scaff2181g030872<br>M.Arenaria_Scaff2148g030567<br>M.Arenaria_Scaff1732g026388<br>M.Arenaria_Scaff1676g025789<br>M.Arenaria_Scaff1383g022579<br>M.Arenaria_Scaff1268g021215<br>M.Arenaria_Scaff960g017422<br>M.Arenaria_Scaff926g016959<br>M.Arenaria_Scaff844g015877<br>M.Arenaria_Scaff784g015089<br>M.Arenaria_Scaff764g014837<br>M.Arenaria_Scaff428g009667<br>M.Arenaria_Scaff376g008772<br>M.Arenaria_Scaff315g007627<br>M.Arenaria_Scaff294g007206<br>M.Arenaria_Scaff264g006653<br>M.Arenaria_Scaff222g005775<br>M.Arenaria_Scaff190g005057<br>M.Arenaria_Scaff185g004950<br>M.Arenaria_Scaff15g000673<br>M.Arenaria_Scaff5g000249	
M.Arenaria_Scaff16790g089239<br>M.Arenaria_Scaff16266g088255<br>M.Arenaria_Scaff16266g088254<br>M.Arenaria_Scaff16127g087997<br>M.Arenaria_Scaff10108g072980<br>M.Arenaria_Scaff10108g072978<br>M.Arenaria_Scaff10108g072977<br>M.Arenaria_Scaff2148g030566<br>M.Arenaria_Scaff2078g029934<br>M.Arenaria_Scaff2078g029932<br>M.Arenaria_Scaff1521g024144<br>M.Arenaria_Scaff1521g024143<br>M.Arenaria_Scaff1521g024142<br>M.Arenaria_Scaff1513g024063<br>M.Arenaria_Scaff1513g024051<br>M.Arenaria_Scaff16g000713<br>M.Arenaria_Scaff9g000422<br>M.Arenaria_Scaff9g000420<br>M.Arenaria_Scaff9g000419<br>M.Arenaria_Scaff5g000247<br>M.Arenaria_Scaff5g000246<br>M.Arenaria_Scaff5g000241<br>M.Arenaria_Scaff5g000240	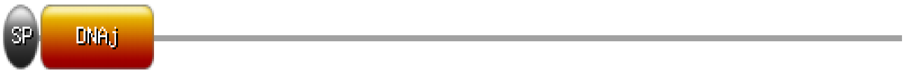
M.Arenaria_Scaff5318g053178<br>M.Arenaria_Scaff5064g051803<br>M.Arenaria_Scaff1296g021554<br>M.Arenaria_Scaff529g011266<br>M.Arenaria_Scaff371g008676	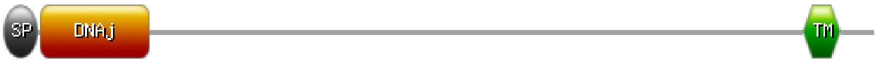
M.Arenaria_Scaff10577g074414<br>M.Arenaria_Scaff7218g062358<br>M.Arenaria_Scaff2273g031734<br>M.Arenaria_Scaff1851g027613<br>M.Arenaria_Scaff1102g019222	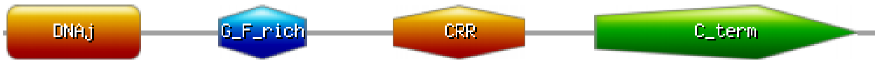
M.Arenaria_Scaff15856g087479<br>M.Arenaria_Scaff14329g084145<br>M.Arenaria_Scaff6657g059801<br>M.Arenaria_Scaff3669g043084<br>M.Arenaria_Scaff3100g038816	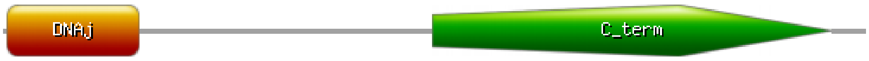
M.Arenaria_Scaff1937g028510<br>M.Arenaria_Scaff193g005120	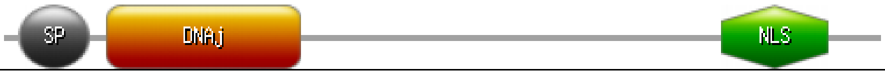

M.Arenaria_Scaff3242g039985
M.Arenaria_Scaff3047g038407
M.Arenaria_Scaff2723g035685
M.Arenaria_Scaff1104g019259

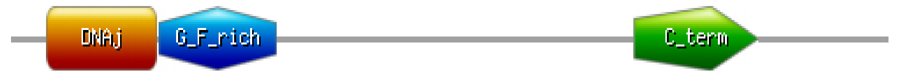

M.Arenaria_Scaff8373g066952
M.Arenaria_Scaff7880g065100
M.Arenaria_Scaff2159g030682
M.Arenaria_Scaff673g013468

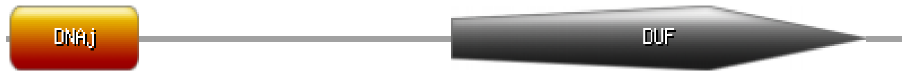

M.Arenaria_Scaff7052g061633
M.Arenaria_Scaff5714g055299
M.Arenaria_Scaff765g014852

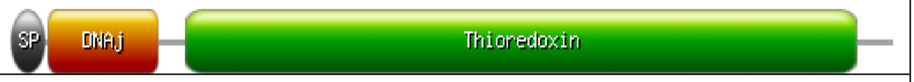

M.Arenaria_Scaff18815g092786
M.Arenaria_Scaff18228g091782
M.Arenaria_Scaff12163g078821
M.Arenaria_Scaff10883g075314
M.Arenaria_Scaff10087g072908
M.Arenaria_Scaff7170g062160
M.Arenaria_Scaff6902g060960
M.Arenaria_Scaff5056g051751
M.Arenaria_Scaff4978g051317
M.Arenaria_Scaff3703g043373
M.Arenaria_Scaff1380g022540
M.Arenaria_Scaff787g015132
M.Arenaria_Scaff378g008807

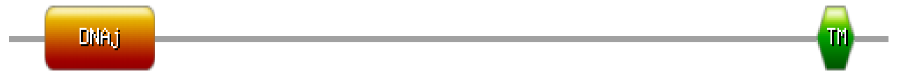

M.Arenaria_Scaff10683g074729
M.Arenaria_Scaff6679g059905
M.Arenaria_Scaff3820g044154

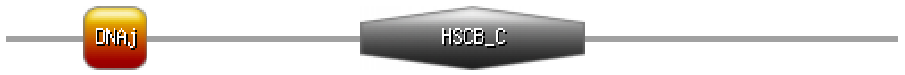

M.Arenaria_Scaff14332g084151
M.Arenaria_Scaff7377g063053

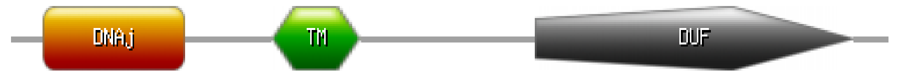

M.Arenaria_Scaff9859g072155
M.Arenaria_Scaff6859g060788
M.Arenaria_Scaff1083g018988
M.Arenaria_Scaff511g010965

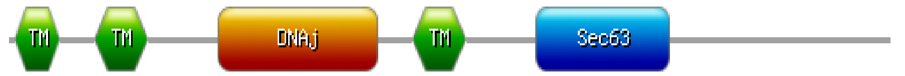

M.Arenaria_Scaff6704g060028
M.Arenaria_Scaff5036g051646
M.Arenaria_Scaff2310g032092

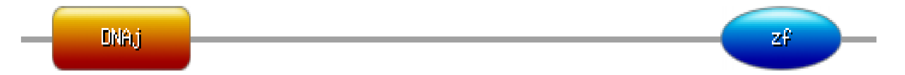

M.Arenaria_Scaff10654g074659
M.Arenaria_Scaff3879g044539
M.Arenaria_Scaff2586g034517
M.Arenaria_Scaff922g016898

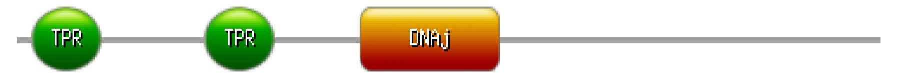

M.Arenaria_Scaff3735g043565
M.Arenaria_Scaff3643g042893

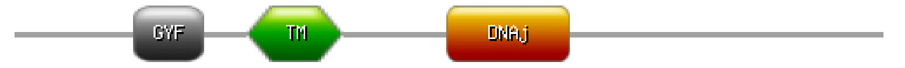

M.Arenaria_Scaff3540g042197

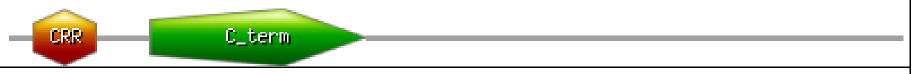

M.Arenaria_Scaff2442g033248

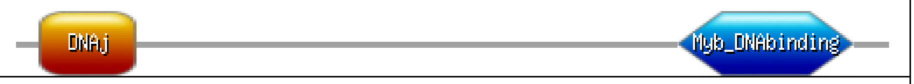

M.Arenaria_Scaff478g010444

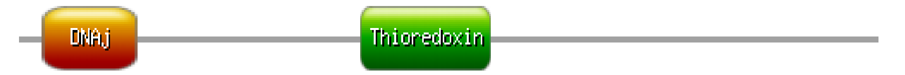

M.Arenaria_Scaff167g004531<br>M.Arenaria_Scaff72g002266	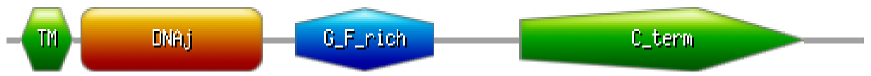
M.Arenaria_Scaff9g000448	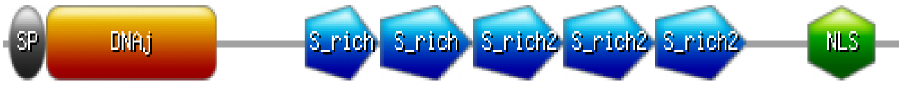
M.Arenaria_Scaff6297g058126	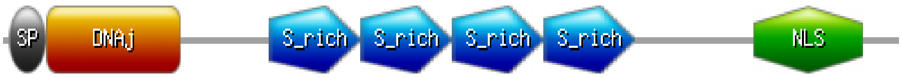
M.Arenaria_Scaff164g004450	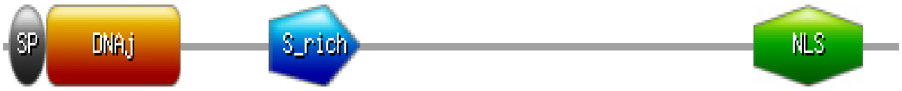

[a] DNAj: Chaperone J-domain of DNAj proteins; TM: Transmembrane motif; SP: signal peptide; NLS: nuclear localization signal; Thioredoxin: Thioredoxin domain; DUF: Domain of unknown function; HSCB_C: Heat shock cognate protein B C-terminal domain; Sec63: Domain associated with the Sec63 complex of yeast; Zf: Zinc finger C2H2 superfamily; TPR: Tetratricopeptide repeat-containing domain; GYF: GYF domain 2; Myb_DNAbinding: SANT/Myb-like domain; G_F_rich: glycine/phenylalanine-rich region; C_rich: Cysteine-rich region; C_term: Carboxy-terminal domain

Supplementary figure 1. Boxplot of the ratio between secreted DnaJ and total DnaJ proteins for 16 nematode species.

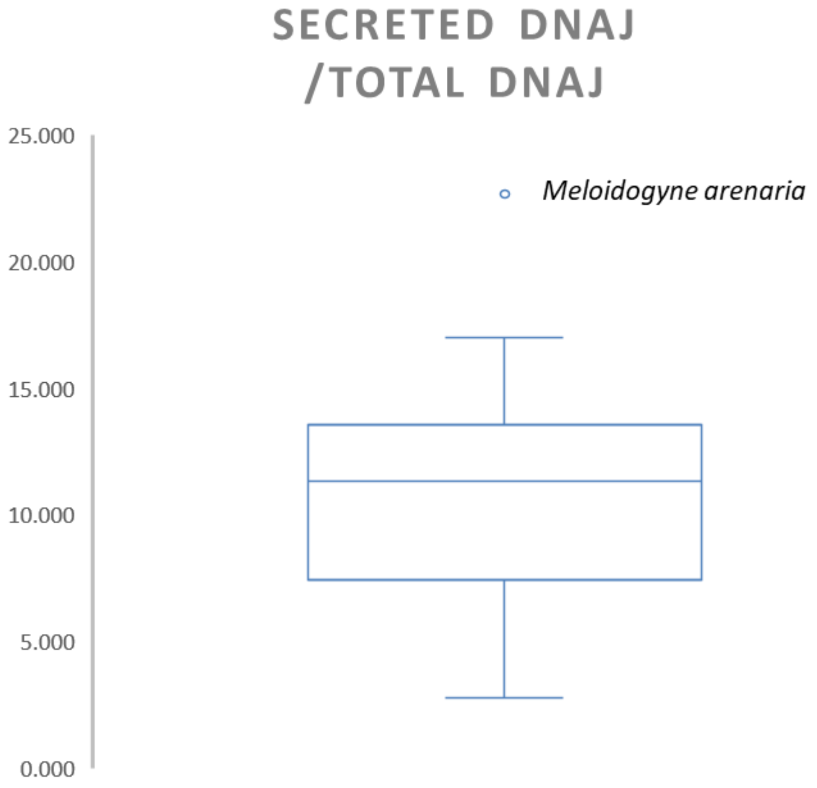

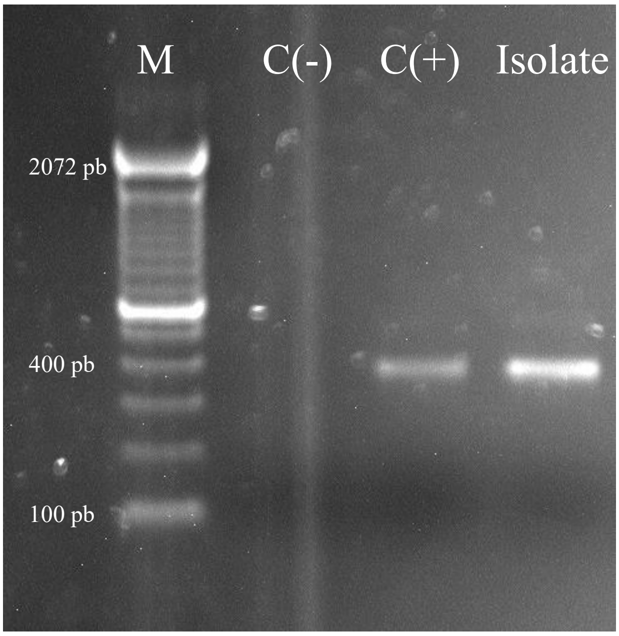

Supplementary figure 2. *Meloidogyne arenaria* SCAR-PCR identification. “M” molecular weight marker 100 bp, “C (-)” negative control (water instead of DNA), “C (+)” positive control (AGROCALIDAD standard DNA for *M. arenaria* identification), and *“*Isolate*”* DNA of collected sample.
